# A single-blind randomized trial on the efficacy of telerehabilitation in post-stroke cognitive impairment. CIPS-TER study: rationale, design and methodology

**DOI:** 10.3389/fstro.2025.1609541

**Published:** 2025-07-23

**Authors:** Eleonora Barucci, Arianna Cavaliere, Eleonora Pavan, Benedetta Formelli, Francesca Cecchi, Cristina Polito, Giulia Salti, Filippo Fratini, Costanza Parenti, Francesca Pescini, Giacomo Redi, Marzia Baldereschi, Antonio Di Carlo, Emilia Salvadori, Anna Poggesi

**Affiliations:** ^1^Neuroscience Section, NEUROFARBA Department, University of Florence, Florence, Italy; ^2^IRCCS Fondazione Don Carlo Gnocchi, Florence, Italy; ^3^Stroke Unit, Careggi University Hospital, Florence, Italy; ^4^Department of Experimental and Clinical Medicine, University of Florence, Florence, Italy; ^5^Institute of Neuroscience, Italian National Research Council, Florence, Italy; ^6^Department of Biomedical and Clinical Sciences, University of Milan, Milan, Italy

**Keywords:** cognitive impairment, stroke, telerehabilitation, cognitive rehabilitation, poststroke cognitive impairment

## Abstract

**Background and aims:**

Cognitive impairment (CI) after stroke is still a neglected consequence compared to other neurological deficits for which rehabilitation pathways are routinely available. Cognitive teleRehabilitation (CTR) represents an emerging approach that has the potential to reduce healthcare costs and potentially reaching many patients. By means of a randomized controlled trial, the aims are to investigate the prevalence of cognitive impairment after stroke and the efficacy of a CTR program in: (a) reducing the risk of CI 6 months after stroke; (b) generalizability of the cognitive training to real life; (c) impact on cognitive performances. In the treated group, feasibility, adherence and appreciation of the CTR will also be evaluated.

**Methods and outcomes:**

The CIPS-TER study is a 2-year prospective, single-blind, randomized clinical trial. One hundred patients with ischemic or hemorrhagic stroke will be enrolled in the study, within 5–21 days after onset, and randomized to treatment or standard care. The CTR program will consist of up to 40 h (8 weeks) of individual treatment based on memory, attention, executive functions, and visuospatial tasks to be autonomously performed with a tablet. The study outcomes will be evaluated at 6-month follow-up visit and will include the diagnosis of cognitive impairment, activities of daily living, quality of life, changes in frailty status and cognitive efficiency.

**Conclusions:**

CIPS-TER study will expand our knowledge on the potential effect of cognitive rehabilitation on future cognitive and functional decline after stroke.

## Background

Stroke is, worldwide, the first cause of disability in adult-older individuals, and the second cause of death and dementia (Leys et al., 2005). Annually, ~16 million of first ever strokes occur in the world, causing 6.3 million deaths (Katan and Luft, 2018; Barbay et al., 2018). Stroke is costly: total cost in Europe is estimated at about €60 billion/year, of which 45% incurred by healthcare systems, 27% for informal care costs, and 22% for loss of productivity (Katan and Luft, 2018; Barbay et al., 2018; Luengo-Fernandez et al., 2020). Future projections suggest an increase in stroke cases, primarily due to the rise in life expectancy (Luengo-Fernandez et al., 2020).

Post-stroke cognitive impairment (PSCI) encompasses all forms of mild to severe cognitive impairment when onset is temporally associated with stroke (El Husseini et al., 2023). The cognitive profile of PSCI is heterogeneous and depends on the type, location, and severity of cerebrovascular lesions. Cognitive hallmarks of stroke-related cognitive impairment include aphasia, neglect, apraxia, and agnosia, and a disconnection syndrome caused by the dysfunction of integrated brain networks and characterized by deficits in information processing speed and executive functions (Shin et al., 2024). Epidemiological data report that ~75% of stroke survivors experience cognitive changes in the acute phase, 47% if we exclude patients with pre-dementia (El Husseini et al., 2023). At a medium-long term cognitive impairment persists in ~50% of patients, a majority fulfilling criteria for mild cognitive disorder (D'Souza et al., 2021). These latter could maximally benefit from cognitive treatment options especially because there are no drug treatments available as today. Cognitive problems after stroke are associated with poor outcomes in terms of recurrence of major vascular events, disability, institutionalization, and death.

Poststroke dementia (PSD) marks the final stage of a spectrum of clinical manifestations of PSCI and refers to all types of dementia following a stroke (Leys et al., 2005; El Husseini et al., 2023). The prevalence of PSD is influenced by the severity of the stroke and the history of recurrent strokes, with PSD occurring less frequently than mild cognitive impairment. Approximately 30% of individuals with a history of stroke develop dementia, with a higher prevalence observed within the first 3 months after stroke compared to 1 year or more later (Leys et al., 2005; El Husseini et al., 2023). Regarding demographic characteristics, studies demonstrate that advancing age and education are key risk factors for PSD (Leys et al., 2005). Additionally, the likelihood of developing PSD is greater in patients who are already dependent before stroke (Leys et al., 2005). Other important factors are neuroimaging features related to pre-existing brain pathologies, independent of index stroke, markers of small vessel diseases, silent infarcts and neurodegenerative markers, such as atrophy and medial-temporal-lobe atrophy (Leys et al., 2005).

Clinical pathways for stroke rehabilitation are inherently influenced by magnitude and heterogeneity of the initial deficits. Severe stroke patients are usually addressed to rehabilitation hospitals where standard programs are primarily devoted to motor, sensory and language deficits. Early cognitive assessment and cognitive rehabilitation are not routinely performed in the acute stroke setting, and there is a lack of an established pathway dedicated to post-stroke cognitive deficits (Suda et al., 2020). Mild stroke patients are frequently discharged at home, thus increasing the risk that persistent cognitive deficits are underdiagnosed and, ultimately, undertreated (Ishiwata et al., 2025). Data on the efficacy of cognitive rehabilitation in both scenarios are needed to identify the characteristics of stroke patients that could maximally benefit of such cognitive interventions. In 2011 a representative group of stroke survivors, carers, and health professionals convened a consensus meeting aimed at compiling the top 10 research priorities of life after stroke, and the most important unsolved question was: “What are the best ways to improve cognition after stroke?” (Pollock et al., 2012, 2014). In line with this, the NICE guidelines for stroke suggest the inclusion of cognitive rehabilitation within rehabilitation pathways, but concluded also that literature does not provide robust evidence about which interventions will be most effective. Despite its social and economic burden is increasingly recognized, cognitive impairment after stroke is still a neglected consequence compared to other neurological deficits for which rehabilitation pathways are generally more available.^1^

At present, cognitive rehabilitation programs remain the most common approach to treat cognitive impairment after stroke. Cognitive rehabilitation stimulates cognitive functions to maximize the patient's degree of autonomy and independence (Viola et al., 2011); it can be provided in a traditional way (face to face, with paper and pencil) or using innovative technology (De cola et al., 2016). In the last decades, advances in Information and Communication Technologies have led to the development of platforms and applications to enable patients' cognitive rehabilitation therapy at home. Telerehabilitation has the potential to provide both individualized rehabilitation treatment and the opportunity of access to rehabilitation services for people who cannot reach the care centers due to logistical and/or functional problems (Lloréns et al., 2015). Standard cognitive rehabilitation has proven to be effective and efficient at improving cognitive performance after stroke. Considering computer-based cognitive training, the results of a meta-analysis by Ye et al. (2022) showed its non-inferiority compared to the traditional method in stroke patients. Emerging evidence indicates that this type of intervention might be effective for individuals with stroke when supervised by an expert and when the program adheres to the neuroplasticity principles (Ye et al., 2022).

Unfortunately, proves on the use of telerehabilitation in stroke patients are based on studies designed to improve motor function rather than cognition. Despite some preliminary evidence to support the effectiveness of computerized cognitive rehabilitation after stroke, high-quality studies focusing on different illness phases, large samples, and various types of intervention software are urgently needed to further improve evidence in this field (Laver et al., 2020).

In recent years, evidence has shown that specific cognitive rehabilitation strategies, especially when structured as goal-oriented training, can significantly improve cognitive outcomes in stroke patients. For instance, a study on subacute stroke patients demonstrated that goal-oriented proprioceptive training not only improved balance, but also stimulated attention functions (Chiaramonte et al., 2024). These findings support the importance of structured, individualized cognitive interventions in the early phases post-stroke.

We present the protocol paper of a randomized control trial aimed at investigating the prevalence of cognitive impairment after stroke and the efficacy of a cognitive telerehabilitation (CTR) program in: (a) reducing the risk of cognitive impairment 6 months after stroke; (b) generalizability of the cognitive training to real life; (c) impact on cognitive performances. In the treated group, feasibility, adherence and appreciation of the CTR will also be evaluated.

## Methods

**Figure 1:**
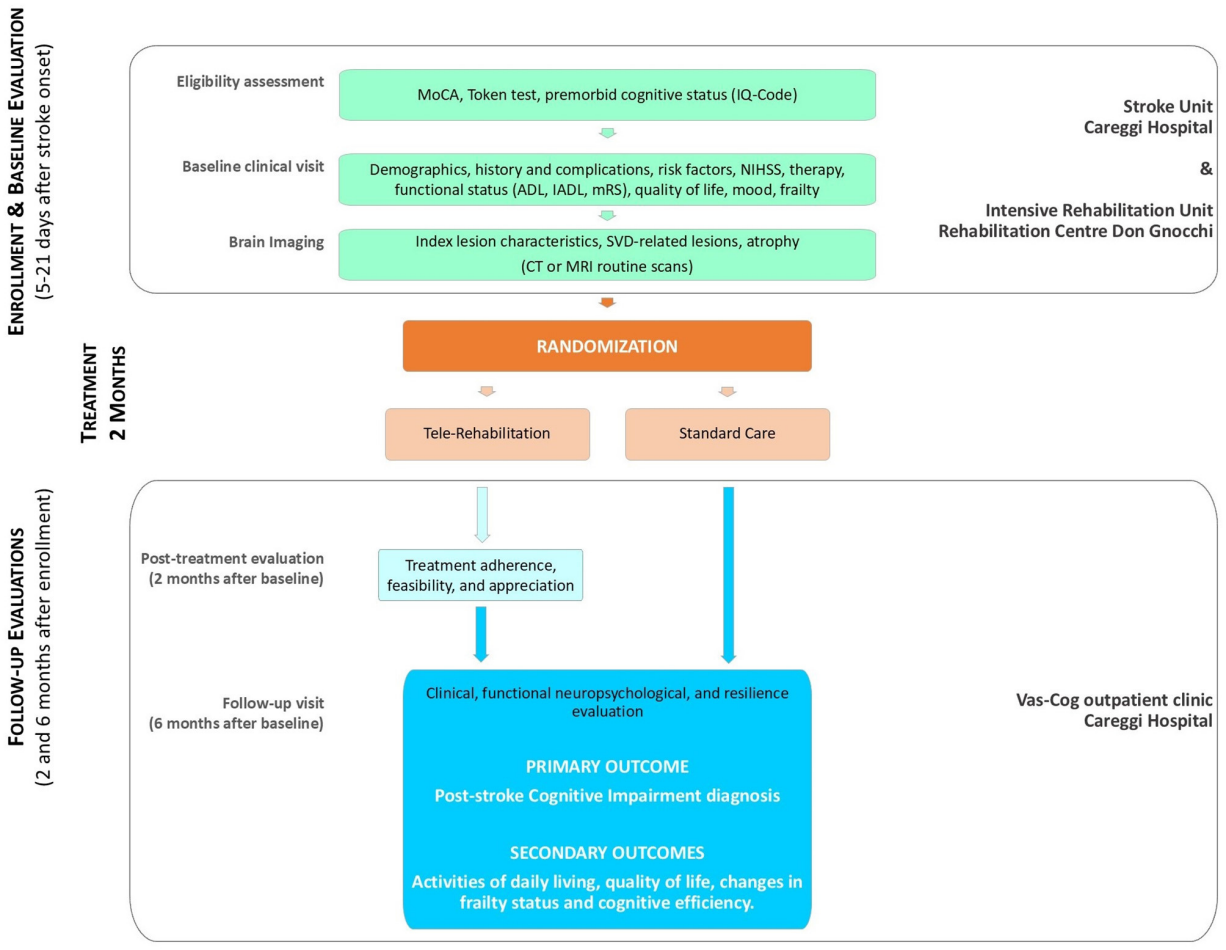
CIPS-TER study experimental design.

CIPS-TER is a 2-year prospective, single-blind, randomized clinical trial aimed at assessing the efficacy of telerehabilitation in stroke patients, started soon after the acute phase of stroke. The experimental design of the study is shown in Figure 1.

The study is carried out at the Stroke Unit of Careggi University Hospital and at General Rehabilitation Unit of IRCCS Don Carlo Gnocchi in Florence, in accordance with the Helsinki Declaration. The study was approved by Local Ethics Committee (25546_spe) and each patient gave a written informed consent.

Patients included in the study were diagnosed with either ischemic or hemorrhagic stroke, regardless of lesion site. Neuroimaging was used to confirm diagnosis.

The study aims at estimating the incidence of post-stroke cognitive impairment in the short and mid-term, and at investigating the efficacy of a CTR program in reducing the risk of CI 6 months after stroke, the generalizability of cognitive training to real life and the impact on cognitive performances. It also aims at evaluating the effect of cognitive training on real-life activities (independence in daily living and quality of life). In the treated group, feasibility, adherence and appreciation of the CTR will also be evaluated.

Inclusion criteria of the study are: ischemic or hemorrhagic stroke (within 5–21 days after onset); age >18 years; evidence of impairment in global cognitive efficiency; NIHSS motor arm item = 0 in at least one arm; fluid Italian speaking; ability and willingness to sign informed consent.

Exclusion criteria are: normal cognitive functioning; pre-existing dementia; severe aphasia; motor deficits in both upper limbs; visual deficits.

All participants diagnosed with ischemic/hemorrhagic stroke are assessed within 5–21 days from onset. Table 1 shows the type of information collected and the instruments applied. Clinical information is retrieved from the clinical records; instruments are administered to patients and caregivers.

**Table 1:** Data collected for screening and baseline evaluation and types of instruments used.

**Study phase**		**Data collection and instruments**
Screening Evaluation	Stroke severity	• NIHSS^*^
	Global cognitive efficiency	• Montreal cognitive assessment (MoCA)^*^ (Nasreddine et al., 2005)
	Verbal comprehension ability	• Token Test in those patients with 1 or 2 at NIHSS language item^*^ (Renzi and Faglioni, 1975)
	Pre-existing cognitive impairment	• Informant questionnaire on cognitive decline in the elderly (IQ-Code)^#^ (Jorm, 2004; van Nieuwkerk et al., 2021)
Baseline Evaluation	Demographics^°^	• Age • Sex • Years of education • Educational degree • Employment status • Profession • Civil status • Living condition
	Vascular risk factors^*^^°^	• Hypertension • Diabetes • Physical Activity • Current smoking habits • Previous smoking habits • Alcohol consumption • Hypercholerestolemia • Hypertriglyceridemia • Atrial fibrillation • Ischemic heart disease • Heart failure • LDL cholesterol assay
	Medical history^°^	• Gait disorders • Thyroid diseases • Migraine/headache • Head injury • Epileptic seizures • Memory disorders • Psychiatric disorders • History of previous stroke
	Home therapy^°^	• Anticoagulants • Antiplatelet agents • Statin
	Secondary prevention therapy adopted in the hospital^°^	• Anticoagulants • Antiplatelet agents • Statin or other treatment
	Neglect	• Star cancellation test^*^ (Halligan and Robertson, 2000; Wilson et al., 1987)
	Apraxia	• Ideomotor apraxia test^*^ (Spinnler and Tognoni, 1987)
	Fragility	• Tilburg frailty indicator (TFI)^*^ (Gobbens and Uchmanowicz, 2021; Mulasso et al., 2016) • Fried criteria (including IPAQ)^*^ (Fried et al., 2001; Mannocci et al., 2010)
	Cognitive reserve	• Cognitive reserve index questionnaire (CRIq)^*^ (Nucci et al., 2012)
	Premorbid functional state	• Activities of daily living (ADL)^#^ (Katz et al., 1963) • Instrumental activities of daily living (IADL)^#^ (Lawton and Brody, 1969) • Modified Rankin scale (mRS)^#^
	Clinical characteristics of index stroke^°^	• Date of stroke • Date of hospitalization • Reason for hospitalization • Lesion type • Pre-stroke mRS • OCSP • TOAST • acute phase treatments • Complications during hospitalization • NIHSS at 24 h • NIHSS at 48 h
	Neuroimaging characteristics of index stroke^°^ Pre-existing brain lesions (Wardlaw et al., 2013; Duering et al., 2023; Scheltens et al., 1992)	• Location • Side • Number of lesions • Leukoencephalopathy • Lacunar infarcts (number, location, side) • Cerebral microbleeds (number, location, side) • Enlarged perivascular spaces (Basal Ganglia, Centrum Semiovale) • Cortical superficial siderosis • Non-lacunar infarcts (number, location, side, vascular territory) • Global cortical atrophy • Mesial temporal atrophy
Discharge Evaluation	Functional state	• Activities of daily living (ADL)^*^ (Katz et al., 1963) • Instrumental activities of daily living (IADL)^*^ (Lawton and Brody, 1969) • Modified Rankin scale (mRS)^*^
	Discharge^°^	• Date of discharge • Discharge destination (home, home with outpatient neurorehabilitation pathway, neurorehabilitation hospital) • NIHSS
	Quality of life	• Stroke adapted sickness impact profile (SA-SIP)^*^ (Buck et al., 2000) • EuroQol scale^*^ (Dorman et al., 1998)
	Mood	• Center for Epidemiologic Studies Depression scale (CES-D)^*^ (Lau et al., 2023)

* Patient interview or evaluation.

° Clinical record.

# Caregiver interview.

For each patient, a caregiver or informant is identified and interviewed to assess the premorbid status. Cognitive premorbid status is evaluated by means of the informant questionnaire on cognitive decline in the elderly (IQ-Code) short form (16 items). IQ-Code explores the changes in daily activities requiring memory and other cognitive abilities observed over the last 10 years. An excellent accuracy of the IQ-CODE for detecting pre-existing dementia in transient ischemic attack and stroke was recently found for an optimal cut-off of >3.48 in a large longitudinal population-based study (Jorm, 2004; van Nieuwkerk et al., 2021). To assess severity of aphasia, we use the language item of the NIHSS according to the following criteria: if the score is 3, the patient is excluded. If the score is 1 or 2, we assess reading capabilities and comprehension. For this latter, we use the Token test (Renzi and Faglioni, 1975), excluding patients with an adjusted score below the 5th centile of the normal population. To be included, patients need to be able to use the tablet autonomously. For this purpose, we exclude patients with severe visual and hearing deficits and those not able to move at least one arm (motor item = 0 in at least 1 arm, not necessarily the dominant one).

Cognitive performances at this stage are assessed by means of Montreal Cognitive Assessment (MoCA). MoCA is a 30-point test recommended by the harmonization standards for the diagnosis and assessment of patients with vascular cognitive impairment, and it covers eight cognitive domains: verbal memory, visuospatial abilities, executive functions, attention, concentration, working memory, language, and orientation to time and place (Nasreddine et al., 2005). The predictive value of MoCA in the acute phase of stroke on the diagnosis of mid-term cognitive impairment is the topic of a previous publication from the proponents (Salvadori et al., 2013). MoCA is a good predictor of mid-term PSCI, independent of other clinical, neurological, and functional characteristics, and a cut-off of 21 is found to have good sensitivity (91%), specificity (76%), positive predictive value (80%), and negative predictive value (89%).

To evaluate cognitive performance, raw scores have been demographically adjusted and converted into an ordinal Five-point scale (equivalent score, ES) using national normative data. Equivalent score is a non-parametric norming method based on percentiles distributions, and scores range varies from 0 to 4: ES = 0 corresponds to an impaired performance (i.e., adjusted score below the outer confidence limit for the 5th centile of the normal population); ES = 1 corresponds to a borderline performance (i.e., adjusted score between the outer and inner confidence limits for the 5th centile of the normal population); and ES = 2–4 represents a normal performance (i.e., adjusted score above the inner confidence limit for the 5th centile of the normal population). When ES methodology is not available, performance was classified according to the reference cut-off, as “normal” when the adjusted score was above the 5th percentile or “abnormal” when the adjusted score was below the 5th percentile in the normal population.

To be included in the study, patients must score ≤ 21 on total MoCA adjusted score (Salvadori et al., 2013) or fail in at least one cognitive domain of MoCA. We consider the inclusion of patients with ES ≤ 1 in at least one cognitive domain according to Aiello et al. (2022).

Through the patient's clinical record, the following information is collected: demographics, medical history, comorbidities, vascular risk factors, home therapy, index stroke clinical and instrumental characteristics (Wardlaw et al., 2013; Duering et al., 2023; Scheltens et al., 1992). A centralized reading of available neuroradiological examinations, such as CT and MRI brain, is performed by an expert vascular neurologist/neuroradiologist.

At baseline, patients undergo a cognitive and mood assessment to investigate: neglect (star cancellation test; Halligan and Robertson, 2000; Wilson et al., 1987), apraxia (ideomotor apraxia test; Spinnler and Tognoni, 1987), fragility [Tilburg Frailty Indicator—TFI (Gobbens and Uchmanowicz, 2021; Mulasso et al., 2016) and Fried criteria (Fried et al., 2001) with IPAQ (Mannocci et al., 2010)] and cognitive reserve (CRI Questionnaire; Nucci et al., 2012). At 7 days after discharge, a telephone interview is conducted to investigate: functional state (ADL, IADL, and mRS; Katz et al., 1963; Lawton and Brody, 1969), quality of life [Stroke adapted sickness impact profile—SA-SIP (Buck et al., 2000) and EuroQol (Dorman et al., 1998)] and mood (CES-D; Lau et al., 2023).

During hospitalization, the caregiver interview involves the investigation of the patient's premorbid functional state (ADL and IADL; Katz et al., 1963; Lawton and Brody, 1969) and the possible presence of cognitive decline before stroke (IQ-Code; Jorm, 2004; van Nieuwkerk et al., 2021).

Stratified block randomization is used to assign participants to the experimental (tele-rehabilitation) or control group (standard care). This method ensures an even distribution of participants across predefined strata and minimizes imbalance in group sizes over time. Participants are stratified based on age, sex, NIHSS and MoCA scores. These variables were selected due to their potential influence on the study outcomes. Blocks of fixed size were generated to ensure that the number of participants in each group remained balanced throughout the enrollment process.

Randomization sequences were created using a computer-based random allocation software by the researchers of the Institute of Neuroscience (Florence) of the Italian National Research Council, who were not involved in participant recruitment or data collection and were in charge of the centralized randomization procedure. To prevent selection bias, randomization sequences are concealed from field workers.

Experimental group: the cognitive telerehabilitation program is based on a medical device, with EU declaration of conformity. The product includes a central workstation, located in the clinic. From the central workstation, an expert psychologist (E.B.), follows patients' performances and weekly adapts the difficulty of the tasks according to patients' performances. During the 2-month treatment, patients' compliance to the training sessions will be recorded.

Each patient randomized in the experimental group receives a tablet for the telerehabilitation program. This foresees sessions of telerehabilitation for 60 min per day, for 5 days a week, for a total of 8 weeks (totally making 40 h of individual treatment). Exercises are meant to work on the main cognitive domains: memory, language, attention, executive functions, visual-spatial and praxis skills. The daily treatment session includes 10-min exercises for each of the six cognitive domains.

Control group (standard care): participants are instructed to have a normal lifestyle and receive conventional treatments based on clinical indications provided by their referring physicians.

Two months after enrollment, at the end of the treatment session, a phone interview will be conducted to investigate satisfaction and gratification regarding CTR.

The endpoints of the study are to detect cognitive deficits in acute post-stroke phase and measure the impact these deficits might have on functional autonomy and quality of life of patients 6 months after stroke. The primary outcome will be the diagnosis of cognitive impairment, including mild cognitive impairment (MCI) and dementia according to the DSM-5 criteria for the diagnosis of mild or major neurocognitive disorders, respectively (American Psychiatric Association, 2022). Secondary endpoints will include assessment of functional status in daily life, quality of life, frailty and cognitive efficiency.

As shown in Table 2, the 6-month follow-up includes a neurological assessment with updates of clinical information, neurological exam and functional evaluation (NIHSS and mRS). Cognitive performances will be assessed by means of a comprehensive multidomain neuropsychological battery. The medical doctor and the neuropsychologist who will perform the follow-up visit will be blinded to treatment group.

**Table 2:** Data collected for the 6-month follow-up visit with types of instruments used.

**Cognitive domain**	**Test**
Global Cognitive Efficiency	• Montreal cognitive assessment (MoCA)^*^ (Siciliano et al., 2019) • Tele-global examination of mental state (Tele-GEMS)^#^ (Montemurro et al., 2023)
Verbal and visual memory	• Rey's Auditory Verbal Learning test (RAVLT)—immediate recall^*^ (Carlesimo et al., 1996) • Rey'S Auditory Verbal Learning test (RAVLT)—delayed recall^*^ (Carlesimo et al., 1996) • Short Story test^*^ (Novelli et al., 1986) • Rey-Osterrieth complex figure (ROCF)—delayed recall^*^ (Caffarra et al., 2002a)
Constructional praxis, spatial planning skills and visual-spatial skills	• Rey-Osterrieth complex figure (ROCF)—copy^*^ (Caffarra et al., 2002a)
Attention and Executive Functions	• Trail Making test—part A^*^ (Giovagnoli et al., 1996) • Visual search^*^ (Della Sala et al., 1992) • Symbol Digit Modalities test (SDMT)^*^ (Nocentini et al., 2006) • Color Word Stroop test^*^ (Caffarra et al., 2002b) • Trail Making test—part B^*^ (Giovagnoli et al., 1996)
Language	• Phonemic verbal fluency^*^ (Costa et al., 2014) • Semantic verbal fluency^*^ (Costa et al., 2014) • Token test^*^ (Renzi and Faglioni, 1975)
Premorbid Intelligence	• Brief Intelligence test^*^ (Colombo et al., 2002)
Neglect	• Star Cancellation test^*^ (Halligan and Robertson, 2000; Wilson et al., 1987)
Apraxia	• Ideomotor Apraxia test^*^ (Spinnler and Tognoni, 1987)
Quality of life	• Stroke adapted sickness impact profile (SA-SIP)^*#^ (Buck et al., 2000) • EuroQol scale^*#^ (Dorman et al., 1998)
Tone and mood	• Center for Epidemiologic Studies Depression scale (CES-D)^*#^ (Lau et al., 2023)
Fragility	• Tilburg frailty indicator (TFI)^*#^ (Gobbens and Uchmanowicz, 2021; Mulasso et al., 2016) • Fried criteria (including IPAQ^#^)^*^ (Fried et al., 2001; Mannocci et al., 2010)
Functional status	• Activities of daily living (ADL)^*#^ (Katz et al., 1963) • Instrumental activities of daily living (IADL)^*#^ (Lawton and Brody, 1969) • Modified Rankin scale (mRS)^*#^

^*^Patient interview or evaluation based on the in-person visit.

^#^Patient interview based on the telephone interview.

The neuropsychological battery will include test to assess global cognitive efficiency (MoCA alternative version; Siciliano et al., 2019), verbal and visual memory [Rey's Auditory Verbal Learning Test—RAVLT (Carlesimo et al., 1996), the Short Story test (Novelli et al., 1986) and Rey-Osterrieth complex figure Delayed-Recall—ROCF DR (Caffarra et al., 2002a)], constructional praxis, spatial planning skills, visual-spatial skills [ROCF copy (Caffarra et al., 2002a)], attention and executive functions [Trail Making Test (Giovagnoli et al., 1996), Visual Search (Della Sala et al., 1992), Symbol Digit Modalities Test (Nocentini et al., 2006) and Color Word Stroop Test (Caffarra et al., 2002b)], language [phonemic and semantic verbal fluency (Costa et al., 2014)], Premorbid Intelligence—IQ (Brief intelligence test—TIB; Colombo et al., 2002), quality of life [SA-SIP (Buck et al., 2000) and EuroQol scale (Dorman et al., 1998)], mood (CES-D; Lau et al., 2023), frailty [Fried criteria (Fried et al., 2001), with IPAQ (Mannocci et al., 2010) and Tilburg Frailty Indicator—TFI (Gobbens and Uchmanowicz, 2021; Mulasso et al., 2016)] and functional status (ADL and IADL; Katz et al., 1963; Lawton and Brody, 1969). Neglect, apraxia and verbal understanding are investigated with the same tests that are included in the baseline assessment.

The study protocol also includes an extensive telephone interview for all patients at the follow-up. By means of the phone interview data on global cognitive efficiency (Tele-GEMS; Montemurro et al., 2023), quality of life [SA-SIP (Buck et al., 2000) and EuroQol scale (Dorman et al., 1998)], mood (CES-D; Lau et al., 2023), frailty [IPAQ (Mannocci et al., 2010) and Tilburg Frailty Indicator—TFI (Gobbens and Uchmanowicz, 2021; Mulasso et al., 2016)], and functional status (ADL and IADL; Katz et al., 1963; Lawton and Brody, 1969) will be collected.

The diagnosis of major and minor neurocognitive disorder will be based on the available clinical assessment, i.e., patients who are not present at the outpatient visit will be classified based on the telephone interview.

### Statistical analysis

The sample size was determined a priori based on data reported in previous studies and reviews on cognitive rehabilitation, along with feasibility and logistical considerations. These included the actual flow of stroke patients admitted to Careggi University Hospital and the Intensive Rehabilitation Unit of IRCCS Don Gnocchi during the planned 12-month enrollment period, along with the availability of dedicated spaces, appropriate settings, and personnel.

Yearly, ~400 stroke patients are admitted to the Stroke Unit of Careggi University Hospital, and ~130 patients are referred to the Intensive Rehabilitation Unit of IRCCS Don Gnocchi. Based on an estimated screening pool of ~550 patients during an enrolment period of 12 months, and accounting for an expected frequency of cognitive deficits in 75% of stroke patients during acute phase (enrolment period), as well as a substantial proportion of ineligible patients and refusals, we estimate to enroll ~100 patients. Eligible patients will be randomized in a 1:1 ratio: 50 will be allocated to the experimental group receiving the telerehabilitation program and 50 to the control group receiving standard care. Both groups will be balanced for key demographic and clinical characteristics. This sample size was determined based on pilot studies and a power analysis to ensure a statistical power of 80% to detect significant differences between the two groups assuming a two-sided alpha level of 0.05.

The data management system will include automatized procedures of data analysis. Specifically, independent sample *t*-tests and chi square tests will be used to compare demographics, vascular risk factors, and global cognitive and functional status, and the results will be available in real time during the whole enrollment period. Stratified block randomization will be employed to balance potential prognostic factors—such as age, gender, MoCA scores, and NIHSS scores—across the two groups, minimizing confounding and enhancing internal validity.

At the conclusion of the 6-month follow-up, primary and secondary outcome analyses will be conducted using SPSS version 28. The analysis will focus on three main objectives:

1) Effectiveness in Reducing Persistent Cognitive Impairment: The efficacy of the telerehabilitation program in reducing the incidence of persistent cognitive impairment at 6 months will be assessed using a chi-square test to compare proportions between groups.
2) Impact on Daily Life Abilities: The effectiveness of the telerehabilitation program in improving patients' functional outcomes will be evaluated through within-group (pre-post) and between-group comparisons. Dependent variables will include functional independence measures, quality-of-life scores, changes in frailty status, and cognitive performance metrics.
3) Predictors of Effectiveness of the telerehabilitation program: Multivariate regression models will be used to identify predictors of telerehabilitation efficacy. Covariates will include demographics (age, sex), cognitive resilience, baseline cognitive and functional abilities, TFI score, index stroke severity, and pre-existing brain structural changes.

The enrolment activities began in April 2024 and are ongoing. Since then, consecutive patients admitted with a diagnosis of ischemic or hemorrhagic stroke are being evaluated for participation in the study.

**Figure 2:**
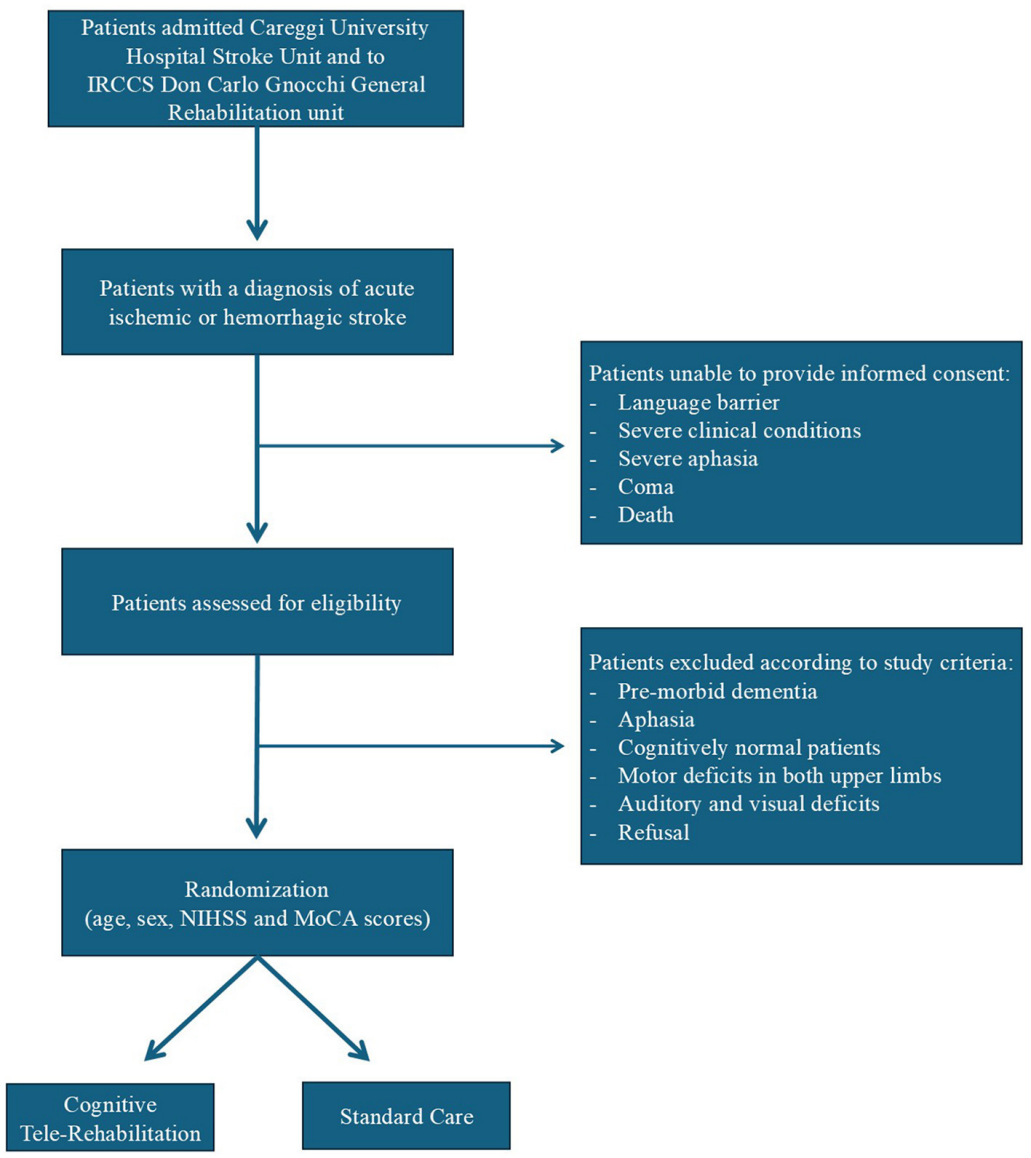
Flow-chart of the CIPS-TER study.

The flow-chart of the study is shown in Figure 2.

## Discussion

Post-stroke cognitive impairment is a very common complication. Available epidemiological data report that about 75% of stroke patients experience cognitive worsening in the acute phase, which for many patients may persist in the medium to long term. These early forms of cognitive impairment could benefit from specific treatments (Ye et al., 2022). In this setting, a randomized controlled clinical trial to evaluate the effectiveness of telerehabilitation could expand knowledge and might bring evidence on feasibility in the acute stroke setting, cost-effectiveness, usability of delivery technologies, and, most importantly, help in the identification of the ideal type of patient who may benefit from such treatment. CIPSTER has been designed with these purposes.

Cognitive rehabilitation interventions focus on restorative and compensatory strategies that aim to restore cognitive function through repeated practice, develop new behaviors to compensate for cognitive deficits, and adapt to these cognitive deficits by reducing the effects of cognitive impairment on functional ability using strategies that minimize demands on attention skills. Post-stroke cognitive rehabilitation interventions have shown some benefits, but evidence is generally limited. Evidence from Cochrane showed that the effectiveness of cognitive rehabilitation for cognitive deficits following stroke remains to be confirmed (Chung et al., 2013; Loetscher et al., 2019; das Nair et al., 2016). Cognitive rehabilitation may improve some specific aspects of attention or executive functions immediately after treatment, but there is no evidence on the persistence of benefits. Improvements after cognitive rehabilitation are often limited, short-term, and dependent on various factors, such as severity of injury, timing of intervention, and type of treatment approach. It is also important to emphasize that tailoring interventions to patients' individual needs is critical to achieve more effective outcomes.

In 2014, Cicerone et al. published recommendations based on preliminary evidence to provide cognitive rehabilitation after stroke for attention deficits, neglect after right-hemisphere stroke, mild memory deficit (compensatory strategies), language deficits after left-hemisphere stroke, deficits in executive functioning, and comprehensive-holistic neuropsychological rehabilitation to reduce cognitive and functional disability after stroke (Cicerone et al., 2019). Telerehabilitation could be a possible alternative to in-person therapy for stroke patients, offering a reasonable model for service delivery, particularly in resource-limited settings (Maresca et al., 2020; Torrisi et al., 2019; Sarfo et al., 2018). The first issue in this regard would be to compare the efficacy between telerehabilitation and traditional “pen-and-paper” rehabilitation. So far, available evidence suggests that the efficacy might be comparable (Ye et al., 2022). Recently, cognitive telerehabilitation has received attention, although, as reported in the Cochrane review by Laver et al. (2020), further investigations are needed as the available evidence suffers from major methodological concerns, mainly small sample size and variability in assessment and treatment options (Laver et al., 2020). There is also growing interest in the use of computerized tools for post-stroke cognitive rehabilitation, which could be very useful in clinical practice for the treatment of specific deficits, potentially enhancing the personalization of interventions (Borsotti et al., 2020). Table 3 shows an overview of main randomized and non-randomized clinical trials on cognitive telerehabilitation in recent years (Nikolaev and Nikolaev, 2022). A notable observation is the consistently small sample size across all studies. In this regard, we propose to randomize a higher number of patients compared to previous studies (Withiel et al., 2019; Gil-Pagés et al., 2018; Faria et al., 2020; Lawson et al., 2020; Isernia et al., 2019; Maresca et al., 2019). CIPSTER is a real-world setting RCT study in the field aspiring to randomize 100 acute stroke patients. However, the relatively modest sample size may still represent a limitation, potentially reducing the statistical power and limiting the generalizability of the findings.

**Table 3:** Overview of main randomized and non-randomized clinical trials on cognitive telerehabilitation in recent years.

**References**	**Type of study**	**Participants**	**Time since stroke**	**Type of cognitive telerehabilitation**	**Duration**	**Intensity**
Withiel et al. (2019)	RCT	65 Memory skills group (MSG, *n* = 24); computerized cognitive training (CCT, *n* = 22); waitlist control participants (WC, *n* = 19)	Chronic	Memory skills group vs. cognitive games online	6 weeks	MSG: 120 min/day 1 times a week CCT: 30 min/day 5 times a week
Gil-Pagés et al. (2018)	RCT	40 Experimental intervention (GNPT^®^, *n* = 20) or sham intervention (ictus.online, *n* = 20)	Chronic	Computerized cognitive telerehabilitation (attention, memory and executive functions exercises) vs. video with quiz	6 weeks	GNTP^®^: 1 h/day 5 times a week Ictus.online: 4 videos of 10 min 5 times a week
Faria et al. (2020)	RCT	32 Reh@City v2.0 (adaptive cognitive training through everyday tasks VR simulations, *n* = 17) vs. Task generator (TG: content equivalent and adaptive paper-and-pencil, *n* = 19)	Chronic	Virtual reality vs. Internet cognitive tasks	4 weeks	12 supervised sessions 3 times a week
Torrisi et al. (2019)	CT	40 Experimental group (*n* = 20) vs. Control group (*n* = 24, standard cognitive training)	Chronic	VR-Evo and VRRS Home Tablet	12 + 12 weeks	VR-Evo 50 min sessions 5 times a week + VRRS Home Tablet 50 min sessions 3 times a week
Lawson et al. (2020)	CT	46 Telehealth delivery condition (*n* = 28) vs. Face-to-face condition (*n* = 18)	Subacute	Zoom videoconferencing	6 weeks	2 h session 1 time a week
Isernia et al. (2019)	CT	45 HEAD rehabilitation in clinic (ClinicHEAD, *n* = 45) + Telerehabilitation at home (HomeHEAD, *n* = 15).	Chronic	Cognitive TeleRehabilitation	4 + 12 weeks	ClinicHEAD: 45 min/day, 3 times a week HomeHEAD: 30–45 min/day 5 times a week
Maresca et al. (2019)	CT	30 Experimental group (*n* = 15) vs. Control group (*n* = 15, paper-pencil tools)	Chronic	Virtual Reality Rehabilitation System-Evo and virtual reality rehabilitation system Home Tablet	24 weeks	50 min/day 5 times a week

RCT, randomized clinical trials; CT, Clinical trials (non-randomized clinical trials).

Another limitation concerns the inclusion and exclusion criteria: usually, in stroke cases, severe strokes and patients with aphasia are excluded, which obviously limits the generalizability of the results. Based on the inclusion and exclusion criteria, we will be able to assess patients with severe cognitive deficits, including mild aphasia, as well as those with very mild deficits, embracing the whole spectrum of post stroke cognitive deficits. By means of the collaboration between a University Hospital Stroke Unit and a Neurorehabilitation ward in rehabilitation Hospital we aspire to test cognitive telerehabilitation in a wide spectrum of stroke severity, in both ischemic and haemorrhagic strokes. This approach will enable the inclusion of patients with a broad spectrum of post-stroke deficits.

A further limitation relates to the risk of non-adherence to treatment. The intensity of the intervention protocol (daily 1-h sessions for 8 weeks) may pose challenges to patient compliance and increase the likelihood of dropout. For this reason, the CIPSTER trial is designed as a feasibility study.

In addition, it is important to acknowledge that the observed improvements in cognitive functions at six-month follow-up might partially reflect the natural course of post-stroke recovery, which tends to be more pronounced in the first few months. Therefore, attributing all the cognitive gains only to the intervention may be confounded by this spontaneous recovery process. The longitudinal evaluation will allow this to be clarified. In fact, the study outcomes will be defined according to standardized criteria based on an extensive neuropsychological and functional evaluation. To limit dropout rates, we also plan to include those patients who will not be able to attend the final visit in person, allowing an extensive phone interview. Of course, at the end, we will compare possible differences between groups.

Last but not least, CIPSTER will shed light on the feasibility of managing cognitive problems also in a very acute phase after stroke, within 3 weeks from stroke onset. Compared with the available evidence, only few studies examined cognitive outcome in this acute phase (Zucchella et al., 2014; Prokopenko et al., 2013), with most focusing on the chronic phase (Ye et al., 2022).

The results of CIPS-TER study may thus contribute to the knowledge on the effects of an early cognitive telerehabilitation on the reduction of persistent cognitive and functional burden in stroke patients, and thus on the potential benefits of including cognitive telerehabilitation as an effective component of post-stroke care pathways after discharge from inpatient care. In line with precision medicine, CIPS-TER project could also help to highlight which clinical features characterize stroke patients who could maximally benefit of such cognitive interventions. Finally, experiences and setups gathered within CIPS-TER project could represent a technical and methodological background for future studies aimed at the development and/or the evaluation of cognitive telerehabilitation systems in stroke patients.

## Data Availability

The raw data supporting the conclusions of this article will be made available by the authors, without undue reservation.

## Footnotes

^1^ https://www.nice.org.uk/guidance/ng236

## Appendix

*List of Participating Centers and Personnel*

Stroke Unit Careggi University Hospital of Florence: Francesco Arba, Maria Lamassa, Benedetta Formelli, Patrizia Nencini, Mascia Nesi, Vanessa Palumbo, Francesca Pescini, Benedetta Piccardi, Anna Poggesi, Cristina Sarti, Laura Tudisco, Arianna Cavaliere, Filippo Fratini, Eleonora Barucci, Giulia Salti.

IRCCS Fondazione Don Carlo Gnocchi: Francesca Cecchi, Cristina Polito, Eleonora Pavan.

Institute of Neuroscience, Italian National Research Council: Antonio Di Carlo, Costanza Parenti, Giacomo Redi, Marzia Baldereschi.
